# Pre-Transplant Serum FTIRS Signatures as Predictive Biomarkers of Early Transient Pancreatic Graft Dysfunction in Simultaneous Pancreas-Kidney Transplantation

**DOI:** 10.3390/life16050780

**Published:** 2026-05-07

**Authors:** Emanuel Vigia, Luís Ramalhete, Rúben Araújo, Sofia Corado, Inês Barros, Beatriz Chumbinho, Ana Nobre, Sofia Carrelha, Paula Pico, Fernando Rodrigues, Miguel Bigotte Vieira, Rita Magriço, Patrícia Cotovio, Fernando Caeiro, Inês Aires, Cecília Silva, Ana Pena, Luís Bicho, Cristina Jorge, Cecília R. C. Calado, Jorge P. Pereira, Aníbal Ferreira, Hugo P. Marques

**Affiliations:** 1 Hepatobiliopancreatic and Transplantation Center, Curry Cabral Hospital, Unidade Local de Saúde de São José, R. da Beneficência 8, 1050-099 Lisbon, Portugal; sofiacorado@gmail.com (S.C.); inesfigueiredodebarros@gmail.com (I.B.); beatriz.chumbinho@gmail.com (B.C.); nobre.ana73@gmail.com (A.N.); sofia_carrelha@hotmail.com (S.C.); anapena64@gmail.com (A.P.); bicho.luis@gmail.com (L.B.); hugoscpm@gmail.com (H.P.M.); 2 Nova Medical School, Faculdade de Ciências Médicas, NMS, FCM, Universidade Nova de Lisboa, 1169-056 Lisbon, Portugal; luis.m.ramalhete@gmail.com (L.R.); rubenalexandredinisaraujo@gmail.com (R.A.); mbigottevieira@gmail.com (M.B.V.); rita.remedios@ulssjose.min-saude.pt (R.M.); patriciacotovio@gmail.com (P.C.); fccaeiro@gmail.com (F.C.); ircaires@gmail.com (I.A.); cecilia.silva77@gmail.com (C.S.); fusilis@gmail.com (J.P.P.); anibalferreira@icloud.com (A.F.); 3 iNOVA4Health—Advancing Precision Medicine, Núcleo de Investigação em Doenças Renais, Nova Medical School, Faculdade de Ciências Médicas, NMS, FCM, Universidade Nova de Lisboa, 1169-056 Lisbon, Portugal; 4 Centro Clínico Académico de Lisboa, 1169-024 Lisbon, Portugal; 5 Blood and Transplantation Center of Lisbon, Instituto Português do Sangue e da Transplantação, Alameda das Linhas de Torres, n◦ 117, 1769-001 Lisbon, Portugal; 6 ISEL—Instituto Superior de Engenharia de Lisboa, Rua Conselheiro Emídio Navarro 1, 1959-007 Lisbon, Portugal; cecilia.calado@isel.pt; 7 Gabinete Coordenador de Colheita e Transplantação, Hospital São José, Unidade Local de Saúde de São José R. José António Serrano, 1150-199 Lisboa, Portugal; paulac.pico@ulssjose.min-saude.pt (P.P.); fernando.rodrigues@ulssjose.min-saude.pt (F.R.); 8 Nephrology, Curry Cabral Hospital, Unidade Local de Saúde de São José, R. da Beneficência 8, 1050-099 Lisbon, Portugal; cristina.jorge2@ulssjose.min-saude.pt; 9 Institute for Bioengineering and Biosciences (iBB), The Associate Laboratory Institute for Health and Bioeconomy-i4HB, Instituto Superior Técnico (IST), Universidade de Lisboa (UL), Av. Rovisco Pais, 1049-001 Lisbon, Portugal

**Keywords:** FTIRS, serum spectroscopy, machine learning, simultaneous pancreas-kidney transplantation, pancreas graft dysfunction, biomarkers, risk stratification, delayed endocrine graft function

## Abstract

Background/Objectives: Early transient endocrine dysfunction after simultaneous pancreas-kidney transplantation (SPK) frequently triggers urgent investigations to exclude thrombosis, pancreatitis, or rejection, yet many recipients recover during the index admission. We tested whether pre-transplant day zero (D0) serum Fourier-transform infrared spectroscopy (FTIRS) captures a biochemical fingerprint associated with a Start&Stop trajectory (initial insulin independence followed by transient dysfunction with recovery). Methods: In a single-center retrospective case-control study nested within 104 consecutive SPK recipients with available D0 serum, 12 Start&Stop cases were matched 1:1 to 12 No-Stop controls. Serum FTIR spectra went through structured quality control and standardized preprocessing. A Naïve Bayes classifier with Fast Correlation-Based Filter (FCBF) feature selection was evaluated using leave-one-out cross-validation (LOOCV) and label-permutation analysis. Results: Under LOOCV, the primary FTIRS model (Savitzky-Golay second derivative; 600–900 and 2800–3400 cm^−1^) achieved excellent discrimination (ROC-AUC 1.00) with accuracy 0.958 and F1 score 0.958. Discrimination collapsed under label permutation (ROC-AUC 0.461), supporting a non-random label-spectrum association. Discriminant information mapped mainly to carbohydrate/glycoprotein-associated bands (~946–1161 cm^−1^), protein structural contributions near the amide III region (~1300 cm^−1^), and lipid/protein stretching modes (~2865–3163 cm^−1^), consistent with a multicomponent systemic biochemical state. Conclusions: In this exploratory matched case-control cohort, pre-transplant D0 serum FTIRS signatures were associated with the subsequent Start&Stop phenotype after SPK. These findings should be interpreted as recipient-side exploratory risk-stratification signals rather than clinically actionable decision tools. Larger multicenter validation in unselected cohorts, with standardized endpoint adjudication, preanalytical control, fully nested model development and inter-instrument harmonization, is required before clinical implementation or population-level risk calibration.

## 1. Introduction

Simultaneous pancreas-kidney (SPK) transplantation remains the most comprehensive treatment for selected patients with type 1 diabetes mellitus and end-stage kidney disease, as it can restore insulin independence while addressing renal failure and improving long-term metabolic stability. Despite these benefits, pancreas transplantation remains clinically demanding because early graft dysfunction may arise from multiple competing causes, including thrombosis, ischemia-reperfusion injury, pancreatitis, infection, surgical complications, drug-related metabolic effects, and rejection [1,2,3,4,5,6,7,8,9,10,11,12,13,14,15,16]. This diagnostic complexity is particularly relevant during the index admission, when early hyperglycemia or renewed insulin requirement may trigger urgent imaging, laboratory investigation, biopsy consideration or empiric therapeutic escalation.

Early pancreas graft dysfunction has been described using heterogeneous definitions, most commonly within the broad framework of delayed endocrine pancreas graft function or pancreas delayed graft function. Traditional definitions generally focus on persistent insulin requirement during a predefined early postoperative window or at hospital discharge [17,18,19]. However, not all early endocrine instability follows a simple failure-to-start pattern. In clinical practice, some recipients initially achieve insulin independence, subsequently develop transient hyperglycemia and/or temporary insulin requirement and then recover endocrine function without irreversible technical graft failure. We operationally define this trajectory as Start&Stop: initial endocrine function, followed by transient interruption, followed by recovery during the index admission.

This trajectory-based phenotype is clinically relevant because it creates uncertainty after apparent initial graft function has already been established. The differential diagnosis includes vascular or surgical complications, ischemia-reperfusion injury, pancreatitis, infection, drug effects and rejection, but the phenotype may also reflect a transient reduction in endocrine reserve or microvascular/metabolic instability rather than permanent graft loss. A tool able to identify recipients at higher risk of this early transient trajectory before transplantation could therefore support more individualized early monitoring, although it should not be used to determine organ acceptance, organ discard or transplant eligibility.

Fourier-transform infrared spectroscopy (FTIRS) offers a label-free method for interrogating the global biochemical composition of biofluids. In serum, FTIRS captures ensemble spectral contributions from proteins, lipids, carbohydrates, glycoproteins, nucleic-acid-related components, and hydration-dependent molecular environments [20,21,22,23]. When combined with machine-learning methods, FTIRS can identify multivariate spectral patterns that may not be apparent from single biochemical measurements. This makes it attractive for exploratory biomarker discovery in settings where the relevant biology is likely to be distributed across several molecular classes rather than represented by one isolated analyte.

In transplantation, the rationale for pre-transplant serum FTIRS is recipient-side rather than donor-side. A D0 serum spectrum does not directly measure donor organ quality. Instead, it may reflect the recipient’s systemic biochemical state before exposure to surgery, preservation injury, reperfusion and immunosuppression. In SPK candidates, this state may incorporate long-standing diabetes, uremia, glycation-related biochemical remodeling, lipoprotein composition, abundant serum protein structure, complement/coagulation-related biology, acute-phase responses and protein hydration or conformational effects. These factors may modulate susceptibility to ischemia-reperfusion injury, endothelial activation, microvascular dysfunction and transient endocrine instability after transplantation.

This study builds on our previous work evaluating early insulin-support trajectories after technically successful SPK transplantation, in which a distinct subgroup showed initial discontinuation of insulin followed by transient resumption before recovery of insulin independence, a pattern termed Start&Stop [24]. Here, we investigated whether pre-transplant D0 serum FTIRS contains a measurable biochemical fingerprint associated with the subsequent Start&Stop phenotype.

Accordingly, the objective of this pilot study was to develop and internally evaluate an FTIRS and machine-learning-based approach for identifying pre-transplant serum spectral signatures associated with Start&Stop after SPK. We used an exploratory matched case-control design nested within a consecutive SPK cohort and interpreted model outputs as internal discriminative scores rather than calibrated population-level risk estimates. The intended clinical concept, if externally validated, would be recipient-side perioperative risk stratification and tailored early post-transplant monitoring, not organ acceptance or transplant eligibility decision-making.

## 2. Materials and Methods

### 2.1. Clinical Question and Endpoint

This case-control study investigated whether D0 (pre-transplant) serum FTIR spectra contain measurable molecular fingerprints associated with a distinctive early post-transplant course in candidates undergoing simultaneous kidney-pancreas transplantation. Start&Stop pancreas graft dysfunction was defined a priori using objective clinical criteria extracted from the medical record. (i) Initial function (Start): insulin independence (no exogenous insulin) sustained for ≥2 consecutive days after transplantation, with fasting glucose within local targets. (ii) Abrupt dysfunction (Stop): reintroduction of exogenous insulin for ≥2 consecutive days and/or persistent hyperglycemia meeting predefined thresholds, occurring after the initial function period. (iii) Recovery (‘Restart’): discontinuation of exogenous insulin for ≥2 consecutive days with return to glucose targets. Two transplant clinicians adjudicated all events independently using a standardized form, blinded to FTIRS/ML results; disagreements were resolved by consensus (or third reviewer). Operational criteria for Start&Stop and adjudication rules are provided.

The analytical objective was to identify spectral biomarkers (discriminative wavenumber regions and multivariate patterns) in pre-transplant serum that are associated with the Start&Stop phenotype and to quantify classification performance under conservative internal validation.

### 2.2. Ethics Approval and Cohort

#### 2.2.1. Ethics Approval and Consent

**Figure 1:**
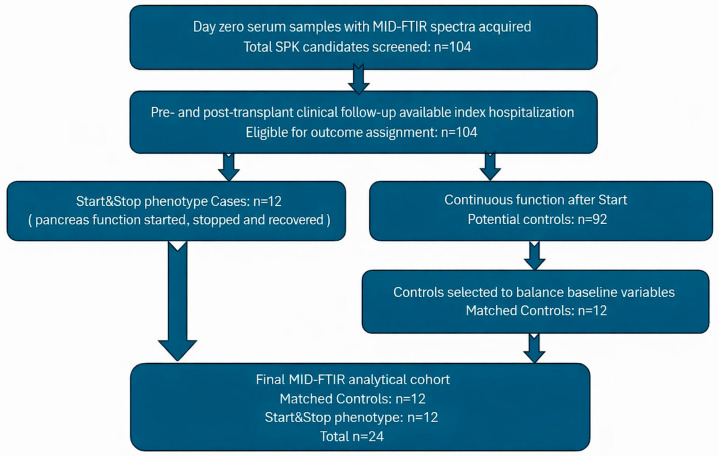
**Patient selection flowchart.** Among 104 consecutive SPK recipients with available D0 serum and interpretable FTIR spectra, 12 fulfilled the prespecified Start&Stop phenotype. A 1:1 baseline-comparable subset of No-Stop recipients was selected for the exploratory matched case-control analysis, yielding a final analytical cohort of 24 recipients.

This retrospective study used routinely collected clinical data and D0 serum samples. Data were extracted and analyzed in a coded (pseudonymized) format, with the re-identification key retained within the institution and access restricted to authorized personnel. The Unidade Local de Saúde de São José Ethics Committee approved the protocol (approval No. 01/2021/CEFCM), and written informed consent for the use of clinical data and stored samples for research purposes was obtained from all participants in accordance with institutional policy and applicable regulations. A study flow diagram (Figure 1) summarizes the number of transplants screened, included, excluded (with reasons), and analyzed in each modeling stage.

#### 2.2.2. Cohort Selection and Matched Case-Control Design

A total of 104 consecutive SPK recipients with available D0 serum were screened. All recipients fulfilling the prespecified Start&Stop phenotype were included (*n* = 12). From the remaining No-Stop recipients (n = 92), a 1:1 subset of controls (n = 12) was selected to create a baseline-comparable exploratory case-control cohort. This matched case-control design was chosen for initial biomarker discovery, with the aim of reducing major imbalances in key recipient, donor, immunological and comorbidity-related variables during this proof-of-concept analysis. Because serum FTIRS captures the global biochemical composition of serum, available baseline comorbidity data were also reviewed as potential spectral confounders, including cardiovascular disease, peripheral vascular disease, hepatic disease, active or recent infection when documented, inflammatory or autoimmune disease, previous malignancy, dyslipidemia, statin therapy and available inflammatory or nutritional laboratory markers recorded in the medical file. However, controls were deliberately selected rather than randomly sampled from the full No-Stop population. Therefore, the analytical cohort should not be interpreted as representative of the overall SPK population and model outputs should be viewed as internal discriminative scores within an enriched matched dataset rather than calibrated estimates of population-level risk. External validation in unselected consecutive SPK cohorts will be required to determine generalizability, absolute risk calibration, and incremental value beyond standard clinical, donor, surgical, immunological and comorbidity-related predictors.

### 2.3. Serum Collection and Handling

Serum was collected on transplant D0 prior to surgery, processed under a standardized institutional workflow, aliquoted to minimize freeze thaw exposure, stored frozen, and thawed once immediately before spectroscopy. All samples were handled under uniform pre-analytical conditions to limit technical variability that could otherwise mimic biological signal in vibrational spectroscopy.

### 2.4. FTIR Spectra Acquisition

Twenty-five μL of serum diluted at 1/10 in Milli-Q^®^ water were plated into a 96-well Si microplate and then dehydrated in a desiccator for 2.5 h under vacuum. Spectra were collected using a FTIRS spectrometer (Vertex 70, Bruker, Germany), equipped with an HTS-XT (Bruker, Germany) accessory. Each spectrum represented 64 coadded scans, acquired in transmission mode between 400 and 4000 cm^−1^, with a resolution of 2 cm^−1^. The first well of the 96-well microplate was left without a sample and the corresponding spectra was used as background, according to the HTS-XT manufacturer.

To mitigate position-dependent and edge effects, sample placement was structured so that wells were alternated between Start&Stop and No-Stop samples, wherever feasible and sample identifiers were masked during preprocessing and modeling. Samples were processed under consistent environmental conditions and instrument configuration to minimize technical variability.

### 2.5. Spectral Quality Assessment

A dedicated quality control (QC) layer was applied before any exploratory or supervised analysis to ensure that downstream separation was not driven by technical artifacts. QC combined quantitative screening with visual review and targeted outlier diagnostics.

Four complementary QC metrics were computed per spectrum:

• Amide I SNR: maximum signal in 1600–1700 cm^−1^ (after baseline handling) divided by noise estimated in 1800–1900 cm^−1^, with a predefined fallback noise window (2000–2200 cm^−1^) when needed.
• Spike burden: spike events quantified in 900–1800 cm^−1^ using the first difference and a robust dispersion estimate Median Absolute Deviation (MAD), counting narrow artifacts exceeding |z| > 6.
• Global spectral coherence (cosine similarity): cosine similarity between each spectrum and the cohort median spectrum computed within the fingerprint window after vector normalization; spectra with unusually low similarity were flagged for review.
• Baseline fraction: baseline dominance summarized as area (baseline)/area (raw) (%) within the fingerprint region, providing a compact index of baseline drift relative to informative structure.

QC metrics were inspected for outliers and interpreted together (rather than relying on a single cutoff) and any spectrum with an abnormal QC profile was reviewed in the context of distance-based diagnostics (heatmaps/clustering) prior to inclusion in modeling.

### 2.6. Spectral Windows and Preprocessing

Analyses focused on spectral windows chosen to preserve biochemical information while limiting regions prone to atmospheric contributions or low interpretability. Two primary window configurations were evaluated: (i) 600–1900 cm^−1^ (fingerprint/amide region) combined with 2800–3400 cm^−1^ (C-H stretching region) and (ii) a reduced fingerprint window 600–900 cm^−1^ combined with 2800–3400 cm^−1^.

Preprocessing was consistent across samples within each pipeline:

• Baseline correction to mitigate low-frequency background trends.
• Vector normalization to reduce multiplicative intensity effects and emphasize spectral shape.
• Savitzky-Golay derivatives (polynomial order 2; 15-point window) to suppress baseline contributions and sharpen overlapping bands; first and second-derivative representations were explored.

Multiple preprocessing variants (raw, rubber-band baseline-corrected, normalized, first derivative, second derivative, and derivative + normalization) were evaluated as sensitivity analyses, and emphasis was placed on representations that produced stable structure without evidence of artifact-driven separation.

### 2.7. Unsupervised Structure Analysis

Unsupervised analyses were used to visualize intrinsic structure, detect residual outliers, and contextualize supervised results. These included cosine-distance heatmaps with hierarchical clustering (Ward-type linkage), metric projections (Multidimensional scaling), and nonlinear visualization (t-SNE) across preprocessing variants.

### 2.8. Supervised Modeling, Validation and Permutation Testing

Supervised learning was used to assess discrimination between Start&Stop and No-Stop using preprocessed spectral features. To minimize information leakage, feature selection and model training were performed within the cross-validation workflow, so that the held-out LOOCV sample did not contribute to feature ranking or classifier fitting in its corresponding training iteration. Feature prioritization was optionally performed using univariate ranking, followed by probabilistic classification using Naïve Bayes.

Given the cohort size (n = 24) and the balanced design, model performance was evaluated with leave-one-out cross-validation (LOOCV). Discrimination was summarized using ROC-AUC as the primary metric, alongside accuracy and class-specific measures (sensitivity/specificity and F1 score) as appropriate.

Score reliability was explored descriptively using a reliability plot based on LOOCV out-of-fold predicted probabilities. Given that the analytical dataset was an enriched matched case-control sample (1:1), no population-level probability calibration or absolute risk interpretation was inferred.

A sensitivity analysis excluding the single recipient with previous transplantation was also planned to evaluate whether this potential confounder disproportionately influenced model performance. The same preprocessing, feature-selection, classification and LOOCV evaluation framework was repeated after exclusion of this case.

### 2.9. Software, Reproducibility, and Computing Environment

All interactive machine-learning workflows (preprocessing variants, feature ranking, unsupervised visualizations, supervised modeling, permutation testing, ROC analysis, and probability calibration) were implemented in Orange Data Mining v3.40.0 (Bioinformatics Lab, University of Ljubljana, Slovenia). Quantitative QC computations and figure generation were supported by custom scripts executed in Python v3.14.0, ensuring that QC metrics and plots could be reproduced deterministically from the exported spectral matrices.

### 2.10. Descriptive Statistics, Baseline Balance and Comorbidities and Clinical Outcomes

Descriptive statistics for baseline clinical, demographic, comorbidity, donor, immunological, and outcome variables were performed in GraphPad Prism 9 (GraphPad Software, San Diego, CA, USA). Continuous variables were summarized as mean ± SD when approximately normally distributed or as median (interquartile range, IQR) when distributions were skewed. Categorical variables were summarized as counts and percentages.

Clinical outcomes, including pancreas graft loss, were compared between groups at 1 year. In addition, time-to-event outcomes were explored using Kaplan-Meier survival analysis, with separate survival curves generated for pancreas graft survival in the Start&Stop and No-Stop groups. Between-group differences in survival curves were assessed using the log-rank test. Given the small sample size and exploratory matched case-control design, outcome comparisons were considered descriptive and hypothesis-generating rather than confirmatory. Two-sided tests were selected according to variable type and distribution, adopting a significance threshold of *p* < 0.05 while avoiding overinterpretation of non-significant findings.

## 3. Results

### 3.1. Study Cohort and Baseline Characteristics

A total of 104 consecutive SPK recipients with available D0 serum generated interpretable FTIR spectra. Among these, 12 recipients fulfilled the prespecified Start&Stop phenotype, defined as initial endocrine function followed by transient interruption and subsequent recovery during the index admission. The remaining 92 recipients did not show this trajectory. For this exploratory biomarker-discovery analysis, 12 No-Stop controls were selected to create a 1:1 baseline-comparable matched case-control cohort. The final analytical dataset therefore comprised 24 recipients: 12 Start&Stop and 12 No-Stop controls.

Baseline characteristics are summarized in Table 1. The matched design reduced major differences in recipient age, sex, BMI, diabetes duration, dialysis exposure, pre-transplant insulin requirement, selected immunological variables, and available donor characteristics. However, because controls were deliberately selected from the larger No-Stop group, this cohort should not be considered representative of the full SPK population. Therefore, the following analyses should be interpreted as exploratory internal discrimination within an enriched matched dataset rather than as population-level risk prediction.

Subsequent analyses focused on whether the pre-transplant D0 serum FTIRS signal contained multivariate biochemical information associated with the Start&Stop trajectory. Where relevant, we assessed whether observed patterns could plausibly arise from technical variability, baseline clinical imbalance, or distributed biological differences captured by the serum spectral fingerprint.

Available baseline comorbidity data were reviewed because systemic conditions may influence serum FTIRS profiles. Major cardiovascular, hepatic, infectious, inflammatory/autoimmune and dyslipidemic conditions were taken into account. No clear clinically relevant imbalance was observed between the Start&Stop and No-Stop groups in the comorbidity variables available for retrospective review. Nevertheless, the small cohort size limits the ability to exclude residual confounding by subclinical inflammatory, metabolic, infectious, or vascular conditions.

**Figure 2:**
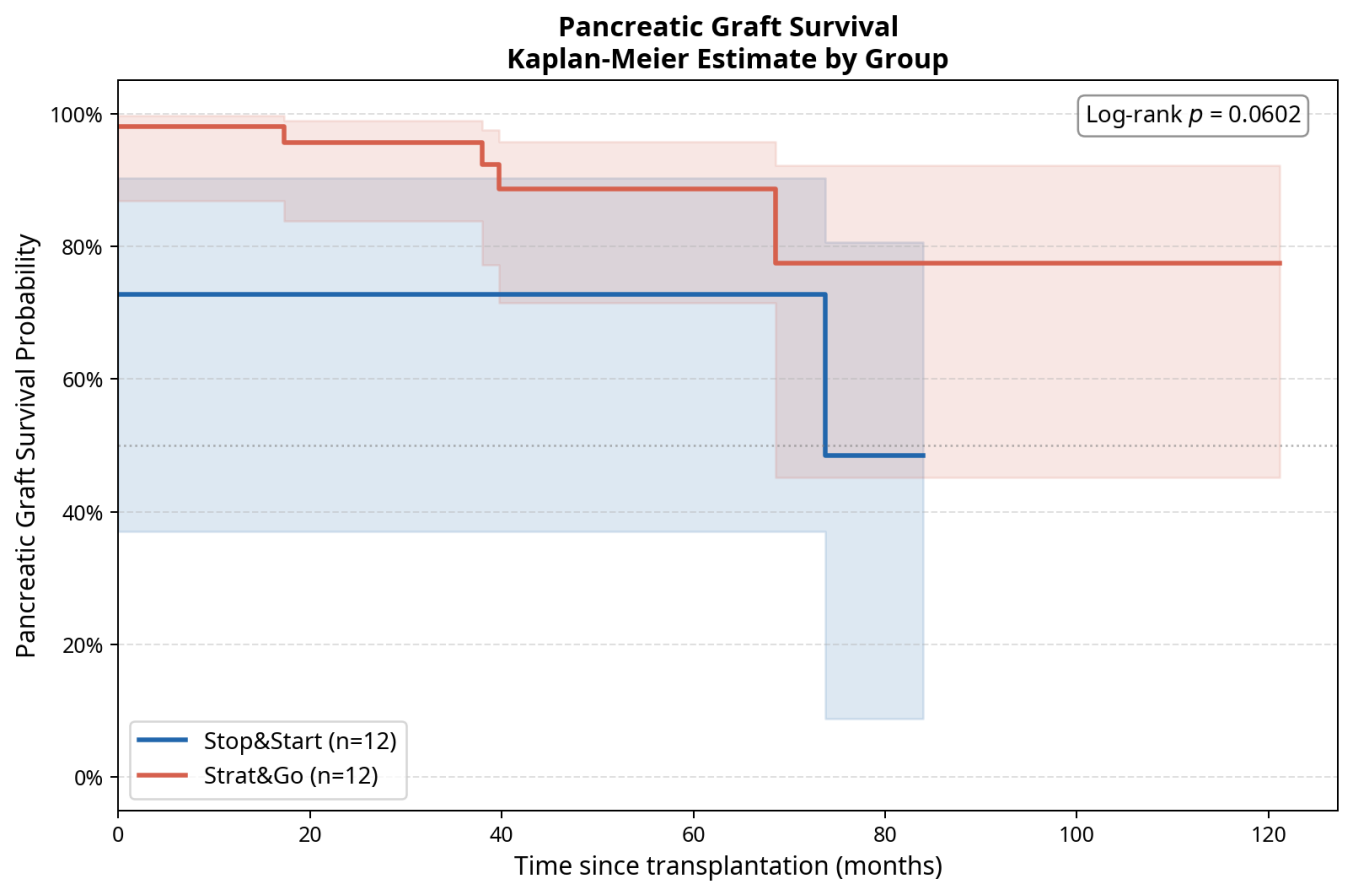
**Pancreas graft survival according to Start&Stop phenotype.** Kaplan-Meier curves comparing pancreas graft survival between Start&Stop recipients (n = 12) and matched No-Stop recipients (n = 12). In the first year, pancreas graft loss occurred in 4/12 Start&Stop recipients and 1/12 No-Stop recipients. Tick marks indicate censored observations. Given the small, matched cohort, this analysis should be interpreted as exploratory and hypothesis-generating.

Pancreas graft survival was further explored using Kaplan-Meier analysis over the first post-transplant year (Figure 2). Pancreas graft loss occurred in 4/12 Start&Stop recipients and 1/12 No-Stop recipients during follow-up. Although this analysis was underpowered and should be interpreted descriptively, the survival curves help contextualize whether the Start&Stop phenotype represents only transient endocrine instability or whether it may identify a subgroup with subsequent risk of irreversible pancreas graft failure.

### 3.2. Quality Assessment of FTIR Spectra

#### 3.2.1. Rationale and QC Strategy

Before any dimensionality reduction or supervised modeling, we performed a structured QC assessment to ensure that:

1. spectra were technically sound (adequate signal-to-noise, limited baseline distortion, and absence of acquisition artifacts), and
2. QC characteristics were not systematically different between the clinical groups (transient pancreatic graft dysfunction, Start&Stop vs. No-Stop group).

This step is essential in infrared biofluid spectroscopy because preprocessing choices can amplify artifacts if the raw spectra are unstable, while genuine biological differences are typically subtle and distributed across bands in the fingerprint and amide regions.

#### 3.2.2. Cohort-Level Spectral Consistency (Visual and Group-Averaged Inspection)

**Figure 3:**
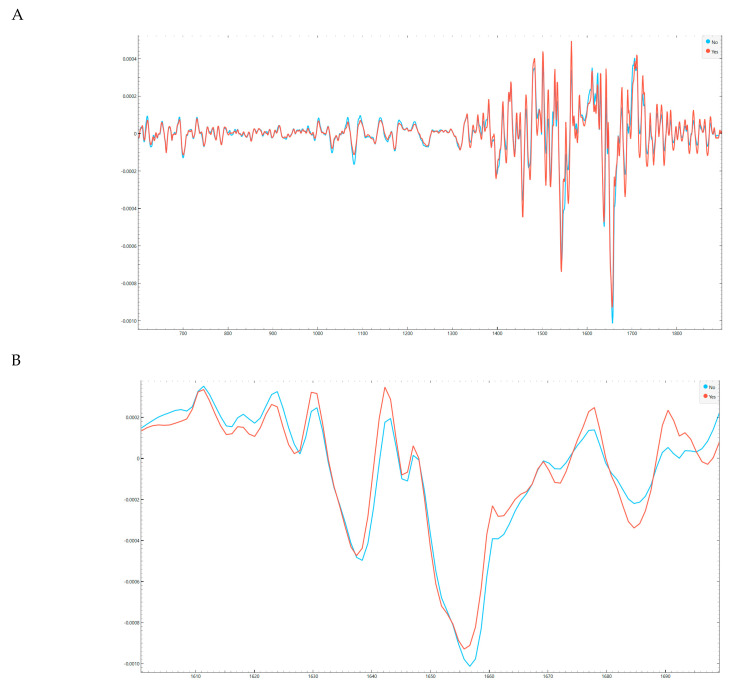
**Group-averaged preprocessed spectra demonstrate high technical consistency.** (**A**) Mean second-derivative (Savitzky-Golay, 15-point window) fingerprint-region spectra by clinical group (Start&Stop vs. No-Stop), highlighting preserved band shapes and absence of gross distortions. (**B**) Mean second-derivative spectra in the protein-dominated region (Amide I vicinity), showing overlapping profiles and no evidence of systematic group-specific acquisition artifacts.

At the cohort level, the mean second-derivative spectra (Savitzky-Golay, 15-point window) demonstrated highly consistent line shapes across the fingerprint and protein-dominated regions, with Start&Stop and No-Stop groups showing broadly overlapping profiles and no gross distortions suggestive of batch effects or systematic sample-handling differences (Figure 3A,B). This is a key prerequisite for downstream feature learning, because mid-IR serum signatures are dominated by proteins (Amide I/II) and mixed biomolecular contributions in the fingerprint region; therefore, technical variability can easily masquerade as “biomarker” signal unless controlled.

#### 3.2.3. Baseline Contribution in the Fingerprint Region

**Figure 4:**
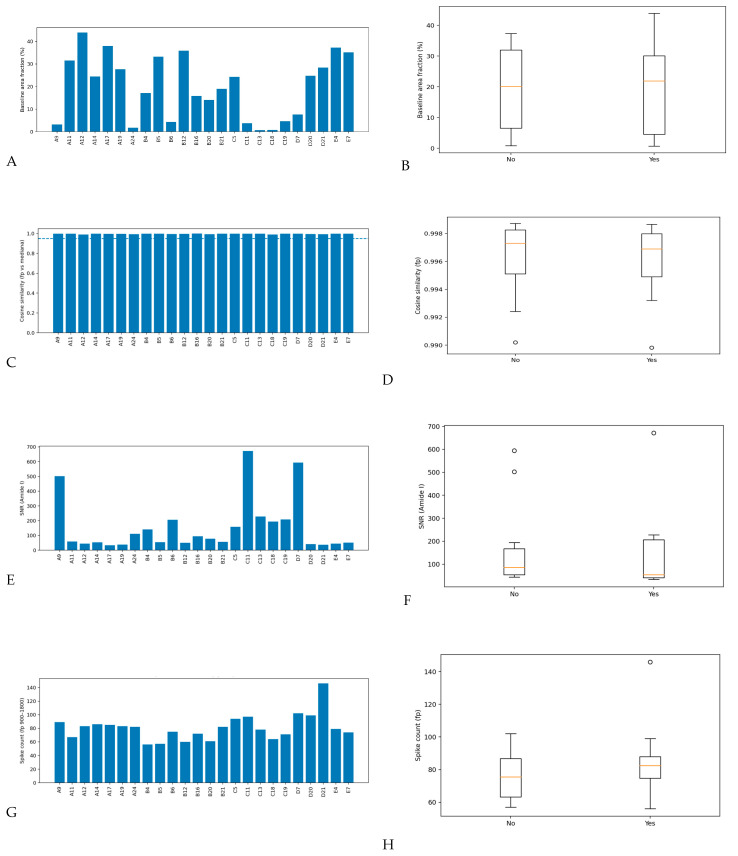
**Quantitative QC metrics for mid-FTIRS serum spectra.** (**A**) Baseline area fraction in the fingerprint region for each sample. (**B**) Baseline fraction summarized by group (boxplots). (**C**) Cosine similarity (fingerprint region) of each spectrum relative to the cohort median spectrum; dashed line indicates a conservative similarity reference threshold used for outlier screening. (**D**) Cosine similarity summarized by group. (**E**) Signal-to-noise ratio estimated in the Amide I region for each sample. (**F**) SNR summarized by group. (**G**) Spike-like artifact burden quantified as the count of robust outliers in the first-difference signal (fingerprint region; MAD-based threshold |z| > 6) per sample. (**H**) Spike count summarized by group.

Baseline distortion in the fingerprint region was quantified as the baseline-area fraction (% of total area). The cohort mean was 19.90 ± 13.98%, with no difference between Start&Stop and No-Stop groups (20.62 ± 14.79% vs. 19.18 ± 13.75%; *p* = 0.807; Table 2; Figure 4A,B). While some spectra showed higher fractions (consistent with film-thickness/scatter variability in dried biofluids), the lack of group imbalance and the use of derivatives/normalization make baseline confounding unlikely.

#### 3.2.4. Spectral Similarity Relative to the Cohort Median (Cosine Similarity)

To detect global outliers, we measured cosine similarity (fingerprint region) between each spectrum and the cohort median spectrum after preprocessing. Cosine similarity values were uniformly high, with an overall median of 0.997 [0.995–0.998], indicating strong coherence of the spectral manifold and minimal evidence of technically aberrant spectra (Table 2; Figure 4C,D). No difference was observed between groups (Mann-Whitney U, *p* = 0.507). This finding supports a stable analytical pipeline and suggests that any group separation seen later in supervised models is more likely to reflect distributed biochemical differences than sporadic technical artifacts.

#### 3.2.5. Signal-to-Noise Ratio in the Amide I Region

Amide I SNR was used as a pragmatic QC metric to confirm adequate information content in protein-dominated bands. SNR was sufficient overall (median 68.4 [48.4–197]) and comparable between Start&Stop and No-Stop groups (53.9 [40.8–207] vs. 85.7 [53.8–167]; *p* = 0.371; Table 2; Figure 4E,F), arguing against noise-driven classification.

#### 3.2.6. Spike/Artifact Burden (Robust Spike Count)

Acquisition artifacts can appear as narrow spikes or abrupt discontinuities that may be exaggerated by derivative preprocessing. We therefore quantified an artifact proxy as the count of spike-like events in the fingerprint region, identified via robust outlier detection on the first-difference signal (MAD-based z scoring; threshold |z| > 6). Spike counts were moderate and broadly comparable across the cohort (overall median 80.5 [70–86.8]; Table 2; Figure 4G,H). There was no evidence of systematic group differences (No: 75.5 [63.3–86.8] vs. Yes: 82.5 [74.8–88]; Mann-Whitney U, *p* = 0.355). One spectrum exhibited a higher spike count than the remainder, but it did not drive group-level separation and was retained to avoid optimistic bias from post hoc exclusions; the downstream pipeline’s rank-based options and cross-validated modeling further reduce sensitivity to single-sample artifacts.

#### 3.2.7. Unsupervised Structure (MDS on Cosine Distance)

**Figure 5:**
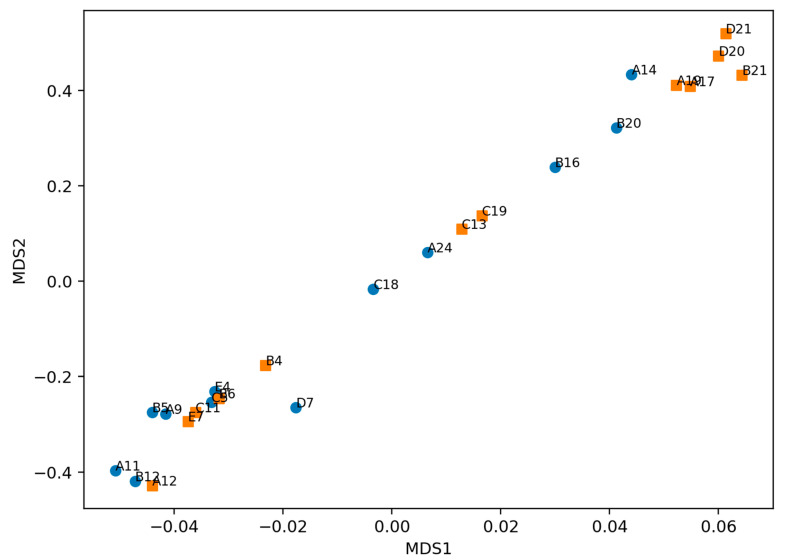
Unsupervised embedding MDS shows no dominant group-driven clustering. MDS using cosine distance computed from second-derivative fingerprint-region representations. Points are labeled by sample ID and colored by clinical group (Start&Stop in orange vs. non-Start&Stop in blue). The overlap between groups supports the absence of major batch effects and suggests that discriminative information, if present, is subtle and distributed across the spectrum.

Finally, we used multidimensional scaling (MDS) on cosine distance (second-derivative fingerprint representation) as a global diagnostic to detect clustering driven by technical effects. The MDS embedding showed substantial overlap between Start&Stop and No-Stop spectra (Figure 5), with no visually dominant separation suggestive of batch effects or acquisition drift. This pattern is consistent with the clinical hypothesis that Start&Stop status is associated with subtle, multiband biochemical differences that are unlikely to be separable by an unsupervised 2D projection alone and instead require supervised learning to integrate weak signals across the spectrum.

#### 3.2.8. Summary: QC Outcomes and Implications for Downstream Modeling

Overall, QC metrics confirm that the final analytical dataset (n = 24; 12 Start&Stop and 12 No-Stop) comprised spectra with stable baselines, adequate-to-high SNR in biologically informative bands, strong concordance with the cohort reference profile, and limited spectral spike contamination. No QC dimension suggested systematic degradation in the Start&Stop group, supporting the interpretation that subsequent class separation and machine-learning performance reflect biological rather than measurement-quality differences.

### 3.3. Baseline FTIRS Spectral Phenotype of Patients with Transient Pancreatic Graft Dysfunction

Group-averaged FTIRS spectra obtained from D0 serum samples revealed a highly consistent biochemical fingerprint across all candidates, with differences between the Start&Stop group (transient pancreatic graft dysfunction) and the No-Stop group emerging primarily as subtle changes in band shape and intensity rather than gross shifts in spectral structure. After standard preprocessing (Savitzky-Golay 2nd derivative, 2nd polynomial, 15-point window) and exclusion of regions dominated by atmospheric contributions, the spectra preserved the serum biochemical profile, including dominant protein-related features in the amide I and II windows and lipid-related CH stretching contributions in the 2800–3000 cm^−1^ region.

When visualized as group means in the fingerprint range, both groups displayed overlapping spectral envelopes, supporting the premise that the cohorts were clinically balanced and not dominated by major baseline metabolic differences. Nonetheless, consistent group-level deviations could be appreciated at discrete spectral positions that correspond to carbohydrate-rich and lipid-associated bands. These deviations were small in absolute magnitude, as expected for serum-based FTIRS biomarkers measured prior to transplantation, but remained reproducible across the 24 samples, suggesting a stable biochemical signal potentially associated with susceptibility to early transient pancreatic graft dysfunction (Figure 3A,B).

In the high-wavenumber region, the Start&Stop group showed modest differences in the CH stretching profile and in the broad water/protein-associated envelope, consistent with subtle baseline variation in lipid packing, protein hydration, or systemic inflammatory state. Taken together, the mean spectra support the biological plausibility of a baseline spectral phenotype that precedes the in-hospital Start&Stop clinical course, while reinforcing that discrimination is likely driven by a small number of informative spectral features rather than by large global compositional changes.

#### 3.3.1. Spectral Structure and Unsupervised Separation

To understand how baseline serum FTIRS spectra organize without using Start&Stop labels, we examined the intrinsic structure of the dataset across the same spectral windows used downstream for modeling. This step is meant to reveal whether the data contains obvious latent grouping, residual batch-like structure, or isolated outliers after the QC and preprocessing decisions described in Section 3.2. Importantly, the absence of a clean, label-aligned partition at this stage would not preclude clinically meaningful discrimination, as FTIRS spectra disease signals are often subtle, distributed, and multivariate, rather than forming separable clusters in low-dimensional embeddings.

##### Hierarchical Clustering and Distance Structure

**Figure 6:**
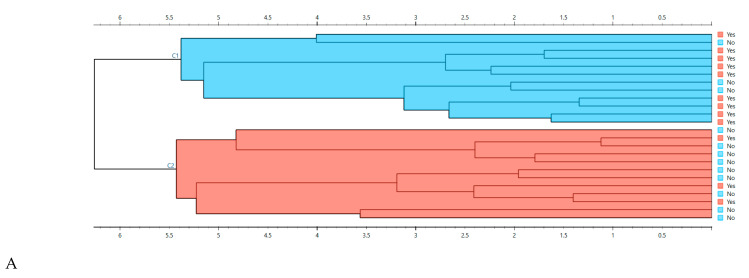

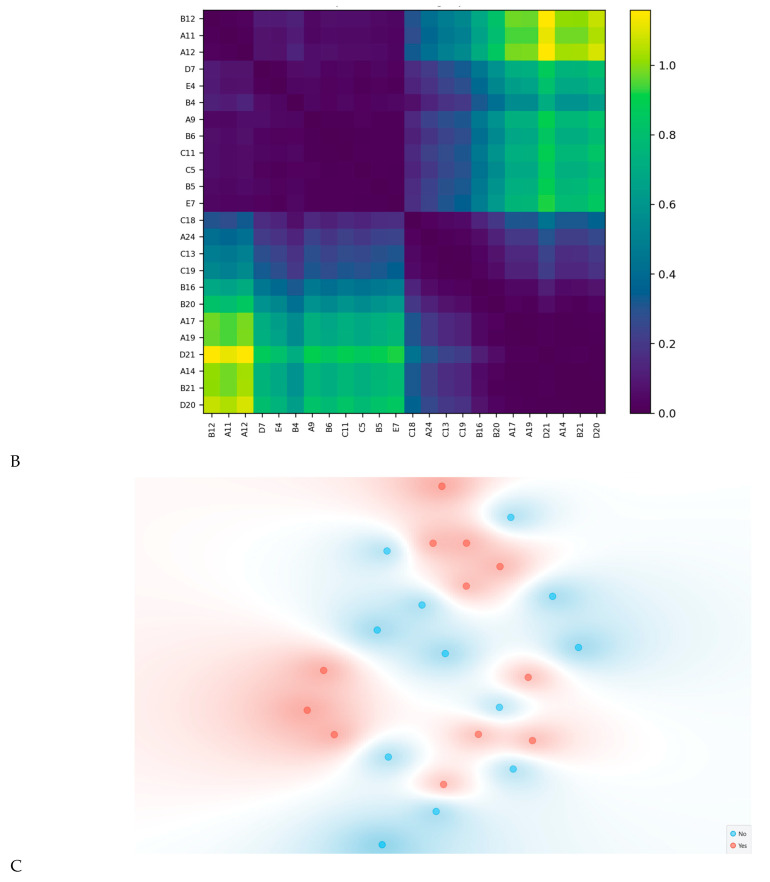
**Unsupervised structure of baseline serum FTIRS spectra.** (**A**) Hierarchical cluster analysis (Ward linkage, cosine distance) revealing two main spectral branches. (**B**) Cosine-distance heatmap ordered by clustering, highlighting block structure with lower within-cluster and higher between-cluster distances. (**C**) t-SNE embedding based on baseline-corrected and vector-normalized spectra (600–1900 and 2800–3400 cm^−1^).

HCA using cosine distance further supported this observation. The dendrogram indicated two major branches, with a visible enrichment of Start&Stop samples within one branch and No-Stop samples within the other (Figure 6A). The corresponding cosine-distance heatmap displayed clear block structure, with lower intra-cluster distances and higher inter-cluster distances (Figure 6B), consistent with meaningful biochemical similarity patterns.

##### Nonlinear Embeddings (t-SNE) Across Preprocessing Variants

A t-SNE embedding built from baseline-corrected and vector-normalized spectra (600–1900 and 2800–3400 cm^−1^) revealed a partially organized manifold, with samples tending to group according to Start&Stop status while still showing a degree of overlap (Figure 6C). This is expected in serum FTIRS data, where disease-related differences can manifest small, coordinated shifts across many wavenumbers. In this case, the pattern indicates that the Start&Stop signature is characterized by a multivariate spectral fingerprint across several biochemical regions, rather than a single dominant peak.

Collectively, these unsupervised analyses demonstrate that baseline serum spectra contain an underlying multivariate organization compatible with Start&Stop class separation, supporting subsequent supervised modeling.

### 3.4. Supervised Discrimination of Start&Stop Using Baseline Serum Spectra

Given the evidence of underlying multivariate organization, we next evaluated whether Start&Stop status could be robustly discriminated using supervised learning applied exclusively to baseline serum spectra.

A Naïve Bayes classifier was selected as the primary model based on repeated internal cross-validation during preprocessing optimization (Table 3). The optimal configuration relied on Savitzky-Golay second derivative filtering (15-point window) combined with vector normalization and ranked feature selection, focusing on the 600–900 and 2800–3400 cm^−1^ windows. These regions capture key biochemical absorptions associated with proteins, lipids, and carbohydrate-related vibrations.

**Figure 7:**
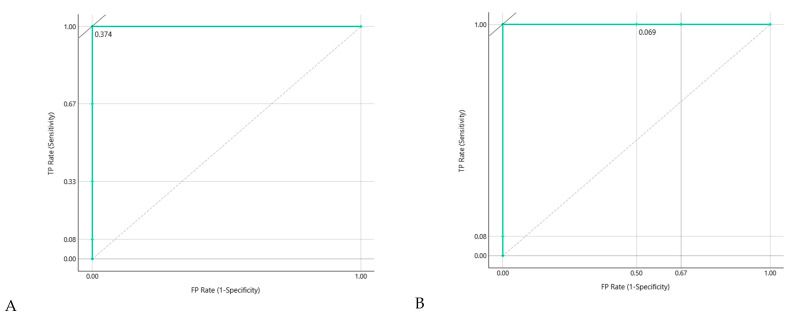
**Receiver operating characteristic (ROC) curve for the primary Naïve Bayes model evaluated by leave-one-out cross-validation (LOOCV).** The model achieved complete class separation (AUC = 1.00), supporting strong discriminative capacity at baseline. (**A**) Target No-Stop. (**B**) Target Start&Stop.

Using LOOCV across all baseline samples, the primary model achieved near-perfect discrimination. The ROC curve demonstrated complete separability between Start&Stop and No-Stop individuals (AUC = 1.00; Figure 7A,B).

Importantly, model interpretability analysis based on ranked spectral contributions identified a compact set of discriminating wavenumbers spanning carbohydrate-associated bands near ~946–1161 cm^−1^, protein-related structure in the amide III region (~1300 cm^−1^), and lipid/protein stretching features in the high wavenumber region (~2865–3163 cm^−1^) (in 2nd derivative plus Unit Vector Normalization) and similar in the case of isolated 2nd derivative (bands near ~620–1000 cm^−1^ and bands near ~2820–3350 cm^−1^). These findings support that Start&Stop status is encoded in a distributed biochemical signature consistent with altered serum composition across multiple macromolecular classes.

#### Sensitivity Analysis Excluding the Retransplant Recipient

Given the rarity of the Start&Stop phenotype and the exploratory nature of this pilot cohort, the retransplant recipient was retained in the primary analysis to avoid further reducing sample size. However, because previous transplantation may influence baseline immunological or inflammatory status, we performed a sensitivity analysis excluding this case. Using the same FTIRS preprocessing and LOOCV modeling framework, the classifier maintained comparable discriminatory performance, indicating that the observed spectral separation was not solely attributable to the retransplant recipient.

### 3.5. Permutation Testing (Internal Validity)

To verify that the observed discrimination was not driven by chance structure, residual batch effects, or inadvertent leakage, we performed a label-permutation test using the same modeling pipeline and evaluation framework as for the primary analysis. In this test, the class labels were randomly permuted (10 permutations), and model performance was recalculated for both the training set and the LOOCV predictions.

**Figure 8:**
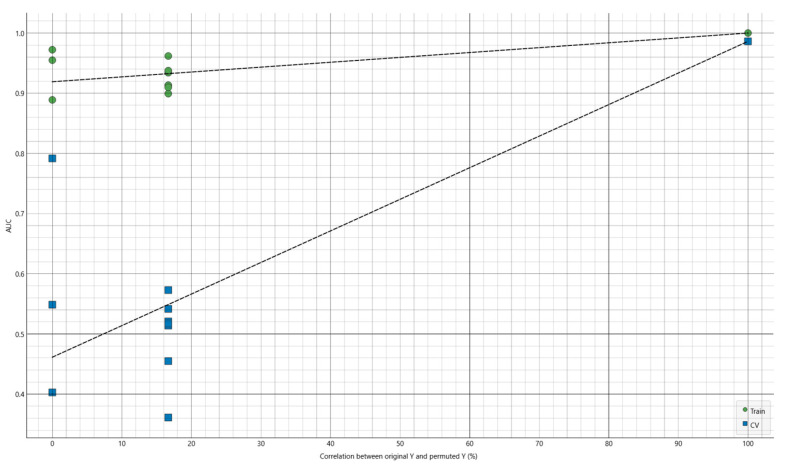
Permutation testing of the primary FTIRS classification model (Start vs. Stop) using baseline serum spectra.

The permutation analysis produced a clear separation between the true-label performance and the null distribution under random labels. With the original labels (correlation = 100%), the model achieved an AUC of 1.00 in training and 0.99 under LOOCV. In contrast, when labels were permuted (correlation = 0%), the corresponding AUC dropped markedly in LOOCV to 0.4613, i.e., near chance level for a binary task, despite the training AUC remaining relatively high (0.9190) (Figure 8). This pattern is expected in small-sample, high-dimensional settings: a flexible classifier can still fit noise in-sample, but it fails to generalize when evaluated out-of-sample (LOOCV). Importantly, none of the permuted runs approached the near-perfect LOOCV performance observed with the correct labels in this experiment, supporting that the discriminative signal captured by the model reflects genuine label-spectrum association rather than an artifact.

### 3.6. Score Reliability

**Figure 9:**
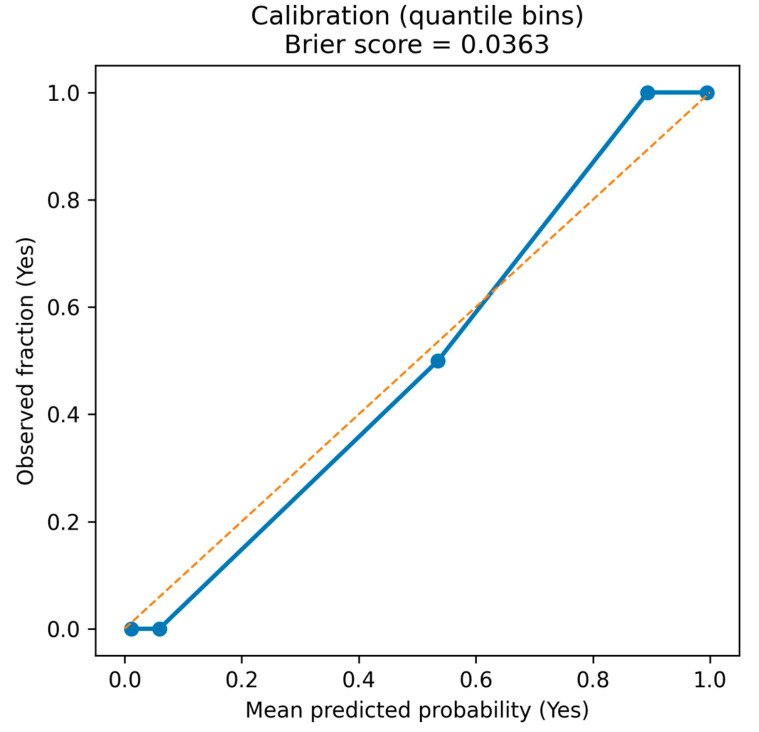
**Calibration plot of LOOCV-predicted probabilities for the primary Naïve Bayes model.** Quantile-binned calibration curve comparing mean predicted probability (x-axis) with the observed fraction of positives (y-axis). The dashed diagonal indicates perfect calibration; rug marks show the distribution of predicted probabilities by outcome class. Shown as an internal descriptive check under an enriched 1:1 case-control design and not as population-level absolute risk calibration.

Beyond discrimination, we performed an internal descriptive check of the probabilistic behavior of the primary model using LOOCV out-of-fold predicted probabilities (Figure 9). The quantile-binned reliability curve showed a monotonic increase in observed event rates from low predicted scores to high predicted scores, consistent with strong class separation. Because this analytical dataset was intentionally enriched to a 1:1 matched case-control ratio, predicted probabilities should be interpreted as internal discriminative scores under the study’s sampling scheme and not as population-level absolute risks; absolute risk calibration would require prevalence-adjusted recalibration and/or validation in a representative cohort. Overall probabilistic accuracy within this matched dataset is summarized by the Brier score (0.0363), which is reported here as an internal accuracy descriptor and should not be extrapolated as a population-risk measure.

### 3.7. Tentative Biochemical Assignments

The discriminant wavenumbers map to biochemical domains commonly observed in serum FTIRS: fingerprint-region modes reflecting carbohydrates/phosphate-containing compounds and protein-associated vibrations, alongside lipid-associated C-H stretching. Specifically, ~945 cm^−1^ is consistent with carbohydrate/phosphate-related vibrations (including glycosidic or phosphate stretching contributions), while ~1161–1299 cm^−1^ may reflect mixed contributions from carbohydrate C-O stretching and protein-associated modes in the fingerprint range. The ~2865 cm^−1^ region lies in the C-H stretching domain and is typically associated with lipid methylene vibrations (symmetric CH_2_ stretch). The high-wavenumber features near ~3145–3163 cm^−1^ may reflect protein/amide-A (N-H stretching) and hydrogen-bonded contributions, potentially capturing differences in protein conformation or hydration environment.

Because serum FTIRS peaks are inherently composite, these assignments should be considered tentative and interpreted as reflecting ensemble biochemical shifts rather than single-molecule specificity.

## 4. Discussion

In this exploratory matched case-control study, we found that pre-transplant D0 serum FTIRS spectra contained a multivariate biochemical fingerprint associated with the subsequent Start&Stop phenotype after SPK transplantation. Using LOOCV within this enriched 1:1 analytical dataset, a Naïve Bayes classifier showed strong internal discrimination between Start&Stop and No-Stop recipients. Label-permutation testing supported that the observed discrimination was not readily reproduced under randomized labels.

These findings should be interpreted cautiously. The matched control design was useful for initial signal detection under reduced baseline clinical heterogeneity, but it may introduce selection bias and overmatching and does not provide population-level calibration. Therefore, the model output should be considered an internal exploratory discriminative score rather than an absolute risk estimate applicable to the general SPK population.

### 4.1. Clinical Interpretation and Novelty of the Start&Stop Phenotype

The Start&Stop phenotype should be distinguished from conventional delayed endocrine pancreas graft function (pDGF). Most pDGF definitions focus on persistent insulin requirement during a defined early postoperative period or at hospital discharge. By contrast, Start&Stop is trajectory-based: the recipient first achieves endocrine function and insulin independence, then develops a transient interruption requiring renewed insulin support and/or showing persistent hyperglycemia, and finally recovers during the index admission. Thus, Start&Stop does not describe a graft that fails to start functioning; rather, it describes an early temporary loss of endocrine stability after initial function has been established. This distinction is clinically relevant because it changes the interpretation of early hyperglycemia. In Start&Stop, renewed insulin requirement occurs after apparent initial graft success, creating uncertainty about whether the episode reflects transient ischemia-reperfusion-related dysfunction, microvascular instability, pancreatitis, drug effects, infection, early immune injury, or evolving technical complications. The phenotype therefore provides a pragmatic clinical endpoint for studying early transient endocrine instability, while acknowledging that it is not yet a standardized international definition [18].

### 4.2. Positioning Within the Contemporary SPK Complication Landscape

Early pancreas graft loss remains dominated by technical failure, most notably thrombosis, and by severe pancreatitis/necrosis, with rejection contributing variably depending on era, surveillance intensity, and case mix. Systematic reviews of SPK cohorts consistently identify donor and recipient factors, procurement/preservation injury and perioperative variables as contributors to early thrombosis risk [5]. Mechanistically, a thrombo-inflammatory milieu may be particularly relevant in pancreas transplantation; recent work has highlighted associations between innate immune activation (including complement activation) and graft thrombosis risk [25]. Against this background, it is plausible that Start&Stop reflects a subclinical instability state in which microvascular dysfunction, inflammatory activation, and/or subthreshold immune injury transiently reduces endocrine function before compensatory recovery.

The present approach should not be directly compared with post-transplant injury biomarkers. For example, donor-derived cell-free DNA addresses a different clinical question and time point, namely the assessment of established graft injury or rejection after transplantation. By contrast, D0 serum FTIRS in this study was explored as a pre-transplant recipient-side signal associated with subsequent early transient endocrine instability. These approaches are therefore different in timing, purpose, and clinical interpretation [26,27].

### 4.3. Biochemical Meaning of the Discriminant Spectral Regions

The model’s discriminative information was concentrated in a limited set of spectral regions, including carbohydrate/phosphate-associated modes in the fingerprint region (~946–1161 cm^−1^), protein-related contributions around the amide III region (~1300 cm^−1^), and lipid/protein C-H and N-H stretching features in the high-wavenumber domain (~2865–3163 cm^−1^). Because serum FTIRS bands are composite and reflect overlapping contributions from abundant circulating biomolecules, these assignments should be interpreted cautiously. Rather than indicating a single molecular pathway, the spectral pattern most likely reflects a broad recipient-side biochemical profile involving glycation-related structures, serum proteins and glycoproteins, lipoproteins, lipid composition, hydration-dependent protein conformation, and possibly acute-phase, complement, or coagulation-related biology.

A plausible interpretation is that the D0 FTIRS signal captures the recipient’s systemic biochemical state before exposure to transplantation-related stress. In SPK candidates, this baseline state may be shaped by long-standing diabetes, uremia, metabolic control, chronic vascular disease burden, protein remodeling, and lipoprotein composition. These factors are not necessarily direct causes of the Start&Stop phenotype; however, they may influence susceptibility to perioperative ischemia-reperfusion injury, endothelial activation, microvascular dysfunction, inflammatory amplification, and transient impairment of endocrine graft function.

This interpretation does not exclude the major contribution of donor organ quality, preservation injury, surgical factors, perioperative hemodynamics, immunological risk, infection, or drug effects. Instead, FTIRS should be viewed as one potential recipient-side biochemical layer within a multifactorial process. The term “systemic biochemical state” is used deliberately: FTIRS does not identify specific cytokines, inflammatory mediators, or single biomarkers. It provides an integrated spectral fingerprint of serum composition. Therefore, the discriminant pattern should be considered a hypothesis-generating signal of recipient biological vulnerability, not evidence of one specific inflammatory mechanism.

Of note, similar protein- and carbohydrate-rich spectral domains have been reported in renal transplantation FTIRS studies using serum fingerprints to classify biopsy-proven rejection phenotypes with machine-learning models [28,29]. Although the organ, timing, and clinical endpoint differ, this overlap supports the broader concept that circulating biochemical remodeling associated with immune activation and tissue injury can be detected by vibrational spectroscopy. In pancreas transplantation, we hypothesize that Start&Stop may represent a threshold phenotype: recipients with a more vulnerable pre-transplant biochemical profile may be more likely to develop transient endocrine instability when exposed to early postoperative stress, even when conventional baseline clinical variables appear balanced.

### 4.4. Limitations of the Study

The principal limitation is the small sample size and single-center design. The final analytical dataset included only 24 recipients, including 12 Start&Stop cases. Therefore, high apparent discrimination, including AUC values approaching 1.0, should be interpreted with caution. Although LOOCV and label-permutation testing reduce the likelihood that the findings reflect purely random label structure, they do not replace external validation in independent cohorts.

Second, the matched case-control design may introduce selection bias and overmatching. Controls were deliberately selected from a larger No-Stop population to reduce major baseline imbalances during this initial biomarker-discovery analysis. This approach may improve internal comparability but limits generalizability and prevents interpretation of the model as a calibrated population-level risk tool. Future studies should validate the FTIRS classifier in unselected consecutive SPK cohorts and test whether it adds incremental value beyond clinical, donor, surgical, preservation, and immunological predictors.

Third, one recipient in the Start&Stop group had a previous transplant. Previous transplantation may influence immunological history, systemic inflammation, and serum biochemical composition. We therefore [performed a sensitivity analysis excluding this patient, which showed…/acknowledge this as a potential confounder]. Future studies should predefine whether retransplant recipients are excluded, stratified, or modeled separately.

Fourth, comorbidities may affect serum FTIRS profiles. Because FTIRS captures global serum biochemical composition, cardiovascular disease, hepatic dysfunction, infection, chronic inflammation, dyslipidemia, hypoalbuminemia, and other systemic conditions may influence protein-, lipid-, carbohydrate-, and glycoprotein-associated spectral regions. We reviewed available comorbidity data, but residual confounding by unmeasured or subclinical conditions cannot be excluded.

Fifth, the Start&Stop endpoint, although clinically intuitive and prespecified in this study, is not yet an internationally standardized pancreas transplant endpoint. Broader reproducibility will require prospective application of objective criteria, including insulin trajectories, glucose thresholds, C-peptide dynamics, imaging findings, rejection assessment, infection status, steroid exposure, and perioperative complications.

Finally, clinical implementation of FTIRS would require prospective multicenter validation, standardized sample handling, inter-instrument harmonization, fully nested model development, external testing, and demonstration of incremental clinical utility. Until such validation is available, the present findings should be regarded as hypothesis-generating.

### 4.5. Clinical Implications and Translation Pathway

Despite these limitations, the present results support a plausible recipient-side translation pathway. FTIRS serum profiling is rapid, low-sample-volume, and inherently multiplexed, making it attractive for exploratory risk stratification in transplant programs. However, the findings should not be interpreted as supporting organ acceptance, organ discard, or transplant eligibility decisions. A recipient with a Start&Stop-like spectral signature should not be denied transplantation on this basis.

If externally validated, a D0 FTIRS classification score could be used to tailor early post-transplant monitoring rather than to decide whether transplantation should proceed. Potential applications may include intensified glucose surveillance, earlier reassessment when hyperglycemia occurs, lower thresholds for targeted vascular imaging if dysfunction emerges, and earlier integration of complementary post-transplant injury biomarkers or clinical review. In this framework, FTIRS would act as a recipient-side warning signal for early transient instability, not as a stand-alone diagnostic test or donor-quality assessment tool.

In parallel, mechanistic follow-up linking discriminant spectral regions to targeted proteomic, metabolomic, lipidomic, and inflammatory panels may clarify the biological drivers of the observed signal and support development of hybrid models combining interpretable molecular features with robust statistical learning.

## 5. Conclusions

In this exploratory matched case-control study of SPK recipients, pre-transplant D0 serum FTIRS identified a composite biochemical signature associated with the subsequent Start&Stop phenotype, defined as early transient interruption of pancreas endocrine function followed by recovery. The discriminant spectral regions involved protein-, glycoprotein/carbohydrate-, and lipid-associated domains, suggesting a broad recipient-side systemic biochemical profile rather than a single disease-specific biomarker.

These findings are hypothesis-generating and should not be used to guide donor organ acceptance, organ discard, or transplant eligibility. If externally validated, D0 FTIRS profiling may support recipient-side perioperative risk stratification and tailored early post-transplant monitoring. Prospective multicenter validation in unselected cohorts, with predefined endpoint adjudication, fully nested model development, inter-laboratory harmonization, and demonstration of incremental clinical utility, is required before clinical implementation.

## Figures and Tables

**Table 1:** Baseline characteristics of Start&Stop and No-Stop recipients.

Characteristic	Start&Stop (n = 12)	No-Stop (n = 12)	*p*-Value
**Recipient baseline characteristics**			
Hospital length of stay (days)	26.50 [18.75–39.25]	21.50 [16.75–28.75]	0.355
Recipient age at transplantation (years)	34.92 ± 6.32	37.67 ± 8.75	0.387
Gender, n (%)	F: 7 (58.3%); M: 5 (41.7%)	F: 7 (58.3%); M: 5 (41.7%)	1.000
Body mass index (kg/m^2^)	24.16 ± 4.82	23.02 ± 1.93	0.458
Dialysis modality, n (%)	Hemodialysis: 8 (66.7%) Peritoneal Dialysis: 3 (25.0%) Pre-emptive: 1 (8.3%)	Hemodialysis: 5 (41.7%) Peritoneal Dialysis: 5 (41.7%) Pre-emptive: 2 (16.7%)	0.525
Dialysis time (days)	521.25 ± 360.35	645.58 ± 551.51	0.520
Diabetes duration (days)	8694.42 ± 2129.07	9240.00 ± 2893.51	0.604
Long-term insulin dose (units/day)	26.00 [18.50–44.25]	26.00 [21.00–32.00]	0.926
Previous transplant, n (%)	Yes: 1 (8.3%) No: 11 (91.7%)	No: 12 (100.0%)	1.000
Pre-transplant serum creatinine	6.33 [3.50–6.86]	5.89 [4.16–6.73]	0.817
Pre-transplant HbA1c (%)	7.75 [7.20–8.30]	8.30 [7.38–9.45]	0.338
**Immunological characteristics (pre-transplant)**			
Prior red-cell transfusions (units)	0.00 [0.00–2.25]	0.50 [0.00–2.00]	0.723
vPRA pre-transplant (%)	4.01 [0.00–26.44]	31.19 [12.03–51.42]	0.170
HLA A/B/DR mismatches, n	4.00 [3.00–4.00]	4.50 [3.75–5.00]	0.178
Pre-transplant DSA, n (%)	Yes: 1 (8.3%) No: 11 (91.7%)	Yes: 2 (16.7%) No: 10 (83.3%)	1.000
**Donor characteristics**			
Donor age (years)	40.50 ± 7.39	33.83 ± 12.84	0.137
Donor body mass index (kg/m^2^)	23.95 ± 3.07	24.66 ± 3.86	0.624
Donor ICU length of stay (days)	3.00 [1.00–5.00]	2.00 [1.00–3.50]	0.655
Donor noradrenaline use, n (%)	Yes: 10 (83.3%) No: 2 (16.7%)	Yes: 6 (50.0%) No: 6 (50.0%)	0.193

**Table 2:** Spectral QC metrics by group.

QC Metric	Overall (n = 24)	No-Stop (n = 12)	Start&Stop (n = 12)
Baseline fraction (fingerprint, % area)	19.90 ± 13.98	19.18 ± 13.75	20.62 ± 14.79
Cosine similarity vs. median (fingerprint)	0.997 [0.995–0.998]	0.997 [0.995–0.998]	0.997 [0.995–0.998]
SNR (Amide I)	68.4 [48.4–197]	85.7 [53.8–167]	53.9 [40.8–207]
Spike count (fingerprint, MAD |z| > 6)	80.5 [70–86.8]	75.5 [63.3–86.8]	82.5 [74.8–88]

*Values are mean ± SD for approximately normal distributions; otherwise, median [IQR]. Normality was assessed per group (Shapiro-Wilk), and test choice followed the pre-specified rule: parametric testing only when both groups were consistent with normality.*

**Table 3:** Naïve Bayes performance across rank-based preprocessing variants.

Main Preprocessing	Secondary Preprocessing	AUC	Accuracy	F1	Precision	Recall	Specificity
2nd derivative	No	1.000	0.958	0.958	0.962	0.958	0.958
2nd derivative	Vector Normalization	0.979	0.958	0.958	0.962	0.958	0.958
Atmospheric compensation	No	0.624	0.625	0.608	0.651	0.625	0.625
Vector normalization	No	0.729	0.750	0.748	0.757	0.750	0.750
1st derivative	No	0.910	0.750	0.750	0.750	0.750	0.750
1st derivative	Vector Normalization	0.931	0.833	0.833	0.833	0.833	0.833
Rubber-band correction	No	0.944	0.875	0.875	0.878	0.875	0.875
Rubber-band correction	Vector Normalization	0.757	0.625	0.608	0.651	0.625	0.625

## Data Availability

The data presented in this study is available upon request from the corresponding author.

## Abbreviations

The following abbreviations are used in this manuscript:

AUC	area under the receiver operating characteristic curve;
BMI	body mass index;
CEFCM	Ethics Committee approval code (ULS São José);
CTA	computed tomography angiography;
DGF	delayed graft function;
DSA	donor-specific antibodies;
dd-cfDNA	donor-derived cell-free DNA;
F1	F1 score;
FCBF	Fast Correlation-Based Filter;
FTIRS	Fourier-transform infrared spectroscopy;
HbA1c	glycated hemoglobin;
HCA	hierarchical cluster analysis;
HLA	human leukocyte antigen;
HTS-XT	High-Throughput Screening extension (FTIRS microplate accessory);
ICU	intensive care unit;
IQR	interquartile range;
IRI	ischemia-reperfusion injury;
LOOCV	leave-one-out cross-validation;
MAD	median absolute deviation;
MDS	multidimensional scaling;
ML	machine learning;
PAK	pancreas-after-kidney transplantation;
pDGF	pancreas delayed graft function;
PTA	pancreas transplantation alone;
QC	quality control;
ROC	receiver operating characteristic;
SD	standard deviation;
SNR	signal-to-noise ratio;
SPK	simultaneous pancreas-kidney transplantation
t-SNE	t-distributed stochastic neighbor embedding;
T1DM	type 1 diabetes mellitus;
ULS	Unidade Local de Saúde;
vPRA	virtual panel reactive antibody;
